# A mycotic aortic aneurysm treated by thoracic endovascular aneurysm repair

**DOI:** 10.1093/jscr/rjz288

**Published:** 2019-11-04

**Authors:** Takahiro Tokuda, Mototsugu Tamaki, Hideki Kitamura, Yutaka Koyama, Koshi Sawada, Yasuhiko Kawaguchi, Kazuya Konakano, Yasuhide Okawa

**Affiliations:** Department of Cardiovascular Surgery, Nagoya Heart Center, Nagoya, Japan

## Abstract

An 88-year-old man was admitted with general fatigue. Computed tomography (CT) showed a descending aortic aneurysm. The laboratory data indicated severe infection. Despite negative blood cultures, broad-spectrum intravenous antibiotic therapy was started. Though antibiotic therapy was continued for about 2 weeks, the aneurysm extended 20 mm. Thoracic endovascular aortic repair was performed, and antibiotic therapy was continued for 4 weeks after the procedure, followed by oral antibiotics for 1 year. CT showed regression of the aneurysm 15 months after reconstruction. Antibiotic therapy, preoperatively and postoperatively, is important for a mycotic aortic aneurysm.

## INTRODUCTION

A mycotic aortic aneurysm is a rare but life-threatening condition. Traditional treatment of mycotic aortic aneurysms is open surgical treatment. A case of successful thoracic endovascular aortic repair (TEVAR) for a difficult case is reported.

## CASE REPORT

An 88-year-old man was admitted to another hospital with general fatigue. His laboratory data showed an inflammatory reaction. Four months previously, he was found to have a descending aortic aneurysm (45 mm) on computed tomography (CT). CT showed a descending aortic aneurysm with a diameter of 50 mm. A mycotic aortic aneurysm was suspected, and the patient was transferred to our hospital.

**Figure 1:**
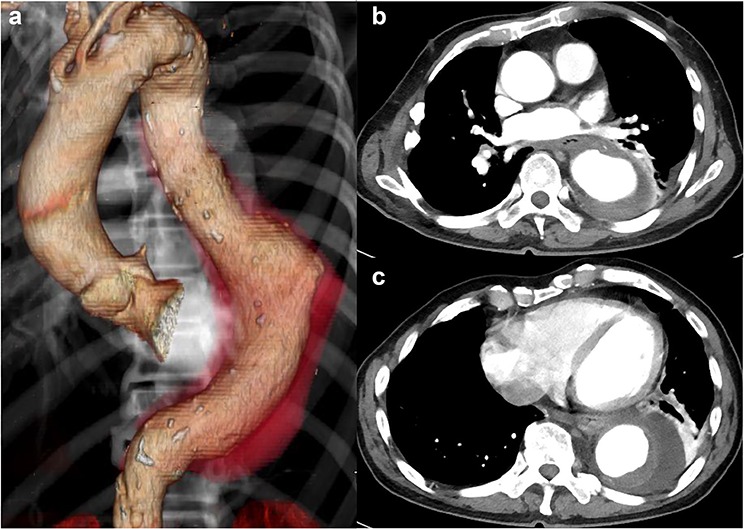
CT shows a descending aortic aneurysm (a), 50 mm in diameter (b), surrounded by an effusion (c).

The patient had no back pain when he came to our hospital. His laboratory data on admission indicated severe infection, i.e. a white blood cell count of 15 080 cells/μL and C-reactive protein of 24.9 mg/dL. CT angiography showed an aneurysm, measuring 50 × 57 mm, with an effusion around it (Fig. 1). Because a mycotic aortic aneurysm was strongly suspected, intravenous antibiotic therapy was started with tazobactam/piperacillin hydrate, despite negative blood cultures.

**Figure 2:**
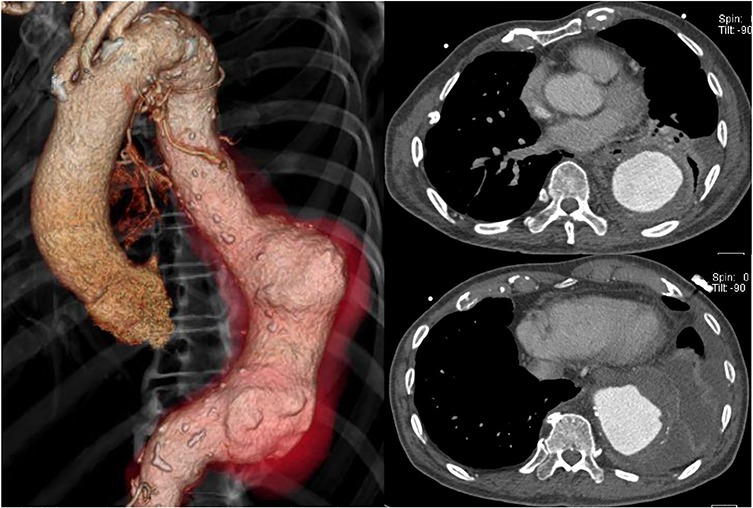
Preoperative CT shows acute expansion of the aneurysm to 70 mm in diameter.

The patient had a fever (38°C) for 4 days after hospitalization, but his temperature dropped to 37°C afterward. His inflammatory reaction gradually improved, but CT angiography showed a descending aortic aneurysm, measuring 70 × 70 mm (Fig. 2). It extended 20 mm in only 2 weeks.

In the present patient, right thoracoplasty had been performed due to tuberculosis, and his breathing function was poor. It was thought that it was very difficult for him to undergo open surgical treatment. TEVAR was performed under local anesthesia to prevent aortic rupture 11 days after admission. Two 37 × 200 mm Conformable stent grafts (W. L. Gore & Associates, Inc., Newark, DE, USA) were deployed through the open right femoral artery approach. No stent endoleaks or dislocation occurred.

Antibiotic therapy continued with the same medication (tazobactam/piperacillin hydrate) for 4 weeks after the procedure. The patient became afebrile with a normal white blood cell count of 5230 cells/μL and C-reactive protein level of 1.42 mg/dL. On postoperative Day 30, he was discharged from hospital without serious complications, but oral antibiotics were continued. Oral antibiotics were continued for 18 months; he was free from antibiotics at the time of writing.

**Figure 3:**
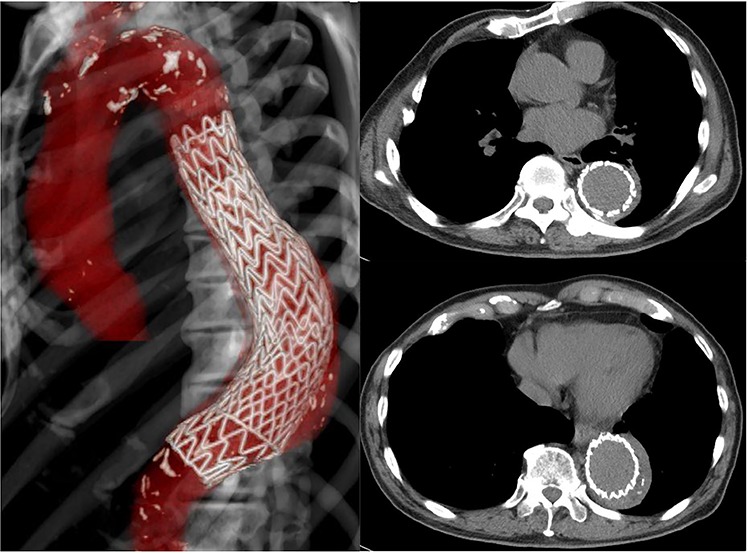
CT 15 months later shows regression of the aneurysm to 53 mm in diameter.

CT 15 months after reconstruction showed regression of the aneurysm, measuring 53 × 53 mm (Fig. 3). He had no further signs of infection.

## DISCUSSION

The most common organisms found to be responsible for mycotic aneurysms are Salmonella spp. and Staphylococcus aureus, but blood cultures are negative in some cases of mycotic aneurysms [1,2]; 77% of patients have at least one positive culture, such as blood, aneurysm wall or bronchial washings [3]. Conversely, no microbes could be isolated from blood and tissue cultures in 25% to 40% of patients [4].

In the present case, the source of the mycobacterial bacilli was unknown. The patient had fever of 37–38°C, his laboratory data indicated severe infection, and the aneurysm extended 20 mm in only 2 weeks. Thus, a diagnosis of mycotic aortic aneurysm was made.

The standard treatment for mycotic aortic aneurysm has been open surgical treatment with debridement of infected tissue and *in situ* repair with an antibiotic-soaked prosthesis followed by long-term antibiotics [1]. As the vascular prosthesis passes into the infected area, the infection may be transmitted. To prevent infection, the omentum or a muscle flap is used to cover the infected field. The mortality for open mycotic aortic aneurysm repair is almost 40% [1,5,6]. As shown by Sörelius *et al.* [7], TEVAR is performed for the treatment of mycotic aortic aneurysm in Sweden. Survival is good: 92% at 1 month and 71% at 5 years. Sedivy *et al.* [8] reported 32 mycotic aortic aneurysm treated with TEVAR. Twenty-six patients (81%) survived for 1 month, and 16 patients (50%) survived for 1 year. Thus, TEVAR may be an alternative to open surgery.

Since the present patient was asymptomatic and CT showed an unruptured mycotic aortic aneurysm, intravenous antibiotics were begun empirically. Despite continued antibiotic treatment for 2 weeks, the aneurysm extended 20 mm, and there was a risk of rupture. His breathing function was poor, and he was very old. It was very difficult for him to undergo open surgical treatment. Thus, it was decided to perform TEVAR and continue the antibiotic treatment for 4 weeks after the procedure. The patient continued to receive oral antibiotics after the intravenous antibiotic therapy.

A lower mortality rate is associated with endovascular treatment of mycotic aortic aneurysms supported with antibiotics from the beginning of treatment, though a stent graft is put in an infected filed [6,9]. Although there is no consensus on the optimal duration of antibiotic therapy, parenteral antibiotics are commonly given for 2–8 weeks after surgery [2]. Antibiotic therapy, preoperatively and postoperatively, is also important for the patient with a mycotic aortic aneurysm [10]. In the present case, intravenous antibiotic therapy was begun preoperatively and continued with the same medication for 4 weeks, and oral antibiotics were continued for 18 months after reconstruction. CT showed regression of the aneurysm 15 months after reconstruction. The patient was alive 30 months after surgery at the time of writing. TEVAR and broad-spectrum antibiotic therapy are important in the treatment of mycotic aortic aneurysms.

In conclusion, endovascular repair of a mycotic aortic aneurysm is an alternative treatment for patients with high operative risk. It is important for the patient to have long-term antibiotic therapy and CT follow-up.

## CONFLICT OF INTEREST STATEMENT

The author(s) declared no potential conflicts of interest with respect to the research, authorship and/or publication of this article.

## FUNDING

None.
